# Using computed tomogram atrial myocardial thickness maps in high‐power short‐duration radiofrequency pulmonary vein isolation: UTMOST AF

**DOI:** 10.1002/joa3.13008

**Published:** 2024-03-01

**Authors:** Taehyun Hwang, Oh‐Seok Kwon, Hee Tae Yu, Song‐Yi Yang, Daehoon Kim, Tae‐Hoon Kim, Jae‐Sun Uhm, Boyoung Joung, Moon‐Hyoung Lee, Chun Hwang, Hui‐Nam Pak, for UTMOST Investigators

**Affiliations:** ^1^ Division of Cardiology, Department of Internal Medicine Yonsei University College of Medicine Seoul Republic of Korea

**Keywords:** atrial wall thickness, catheter ablation, paroxysmal atrial fibrillation

## Abstract

**Background:**

High‐power short‐duration (HPSD) ablation creates wide, shallow lesions using radiofrequency (RF) heating. It is uncertain if adjusting RF energy based on atrial wall thickness provides extra benefits. We studied the safety and effectiveness of tailored HPSD energy based on left atrial (LA) wall thickness (LAWT) for circumferential pulmonary vein isolation (CPVI) in patients with paroxysmal atrial fibrillation (PAF).

**Methods:**

We enrolled 212 patients (68.4% male, mean age: 59.5 ± 11.0 years) and randomly assigned them to two groups: LAWT‐guided CPVI (WT, *n* = 108) and conventional CPVI (control, *n* = 104). Both groups used an open irrigated‐tip deflectable catheter to apply 50 W for 10 s to the posterior LA, while controls used 60 W for 15 s on other LA regions. RF delivery time in WT was titrated (15 s at LAWT > 2.1 mm, 13 s at 1.4–2.1 mm, and 11 s at <1.4 mm) according to the computed tomogram‐myocardial thickness color map.

**Results:**

After a mean follow‐up of 13.4 ± 7.0 months, the WT and control groups showed no significant difference regarding clinical recurrence rate (13.9% vs. 5.8%, respectively; *p* = .061) and major complication rate (4.6% vs. 3.8%, respectively; *p* > .999). The total procedure time, cardioversion rate, and post‐procedural AAD prescription rates did not significantly differ between the groups.

**Conclusions:**

The LAWT‐guided energy titration strategy did not result in improved procedural safety and efficacy compared to the conventional 50–60 W‐HPSD CPVI in patients with PAF.

## Graphical abstract

Overview of the methods and results of our study. The clinical recurrence rate between WT‐guided ablation and control groups did not significantly differ.

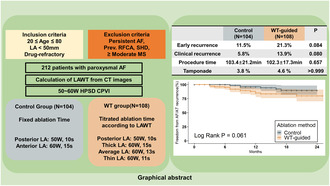

## 1 INTRODUCTION

Catheter ablation is the most effective rhythm control strategy for patients with atrial fibrillation (AF)^1^; however, continuous long‐term recurrence is expected after the procedure.^2^ Pulmonary vein (PV) isolation (PVI) is the cornerstone of AF catheter ablation (AFCA), and PV reconnection is one of the most common mechanisms of AF recurrence after the procedure.^3^ PVI durability can be affected by various factors such as radiofrequency (RF) power^4^; duration of energy delivery^5^; catheter stability^6^; objective physical parameters including ablation index,^7^ or local catheter impedance drop.^8^ Moreover, patient‐specific atrial wall thickness (WT) may be a factor to consider for appropriate transmural lesion generation^9^ as it is vital in evaluating the safety, efficacy, and durability of PVI.^10^ The risk of procedure‐related complications, including pericardial tamponade, increases in elderly females with relatively thinner PV antrums than healthy young males.^11^

Recently, higher power short‐duration (HPSD) RF‐PVI has been widely used, and multiple studies evaluating its efficacy and comparative safety are emerging.^12^ We recently reported no difference in the long‐term efficacy of 60 W HPSD PVI using FlexAbility catheters (Abbott Inc., USA) compared with conventional 35 W RF‐PVI.^13^

Therefore, we explored the efficacy and safety of antral WT‐guided HPSD RF‐PVI by titrating 50–60 W of energy delivered to each point. Based on our previous studies and experience, 15 s of 50–60 W HPSD using FlexAbility was considered the control treatment. We aimed to compare control treatment with WT‐guided ablation, which reduces RF delivery time in thin left atrial (LA) WT (LAWT) areas. We measured and utilized the LAWT using automated customized software (AMBER, LaonMed, Korea) in real time during the AFCA procedures.^14^ We aimed to compare the efficacy and safety of HPSD energy titration according to the LAWT during circumferential PVI (CPVI) in patients with paroxysmal atrial fibrillation (PAF).

## 2 METHODS

### 2.1 Study population and randomization

**FIGURE 1:**
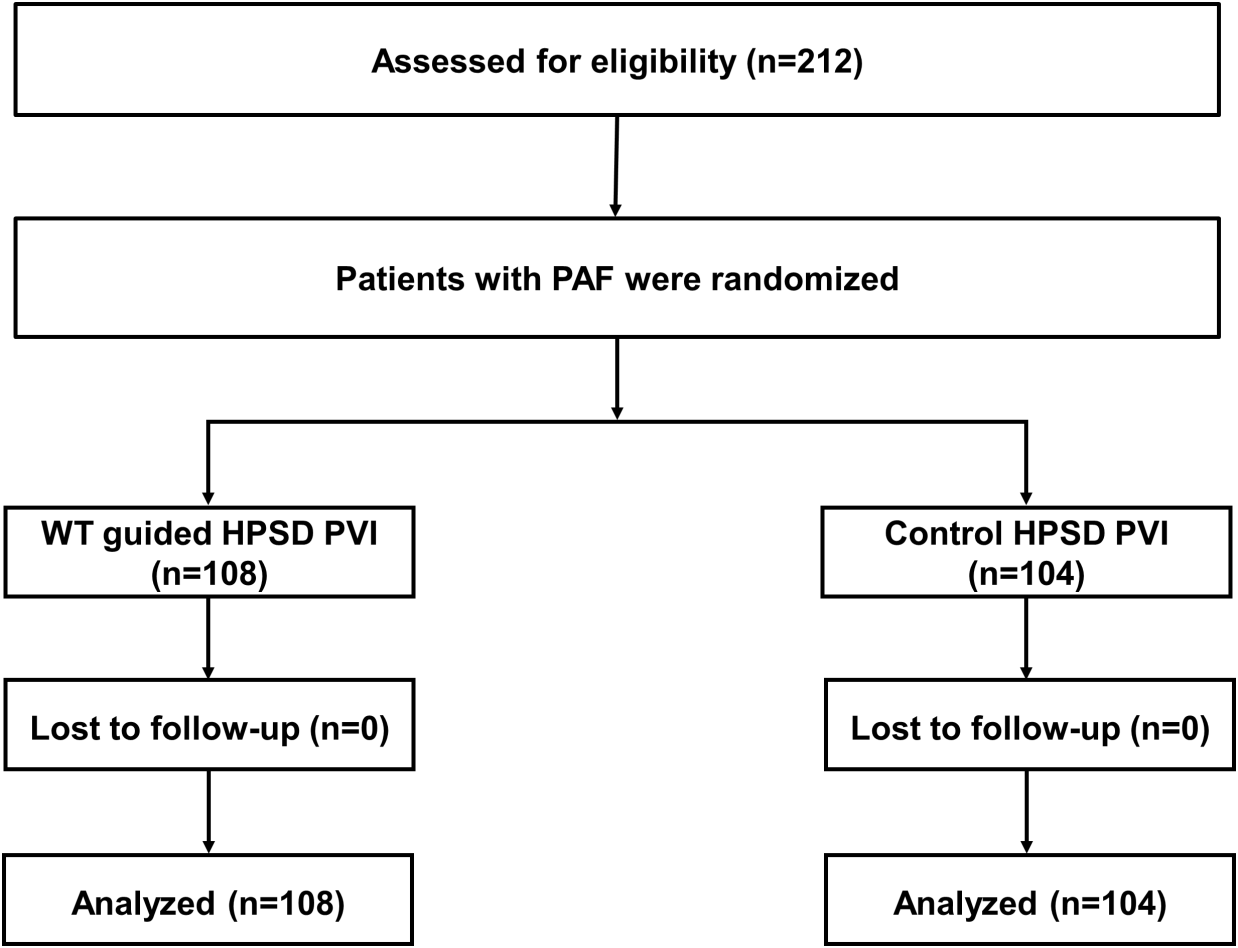
Flow diagram of study population enrollment. HPSD, high‐power short duration; PAF, paroxysmal atrial fibrillation; PVI, pulmonary vein isolation; WT, wall thickness.

This prospective, single‐center, randomized clinical study was performed under the Declarations of Helsinki. Ethical approval was obtained from the institutional review board of Yonsei University, and written informed consent was obtained from all participants before the start of the study (CRIS number: KCT0003747). The supplementary material (Table S1) provides information about the criteria for inclusion and exclusion. This study included patients scheduled for ablation of symptomatic and drug‐refractory PAF. The exclusion criteria were persistent or permanent AF, congenital anomalies that provoked hemodynamic instability, and ineligibility for computed tomography (CT) imaging due to the possibility of kidney damage. To avoid potential bias, we performed randomization using computer‐generated random permutations, and the patients were blinded to the initial allocation (Figure 1).

### 2.2 Preparing LAWT maps from CT images using an automated program

**FIGURE 2:**
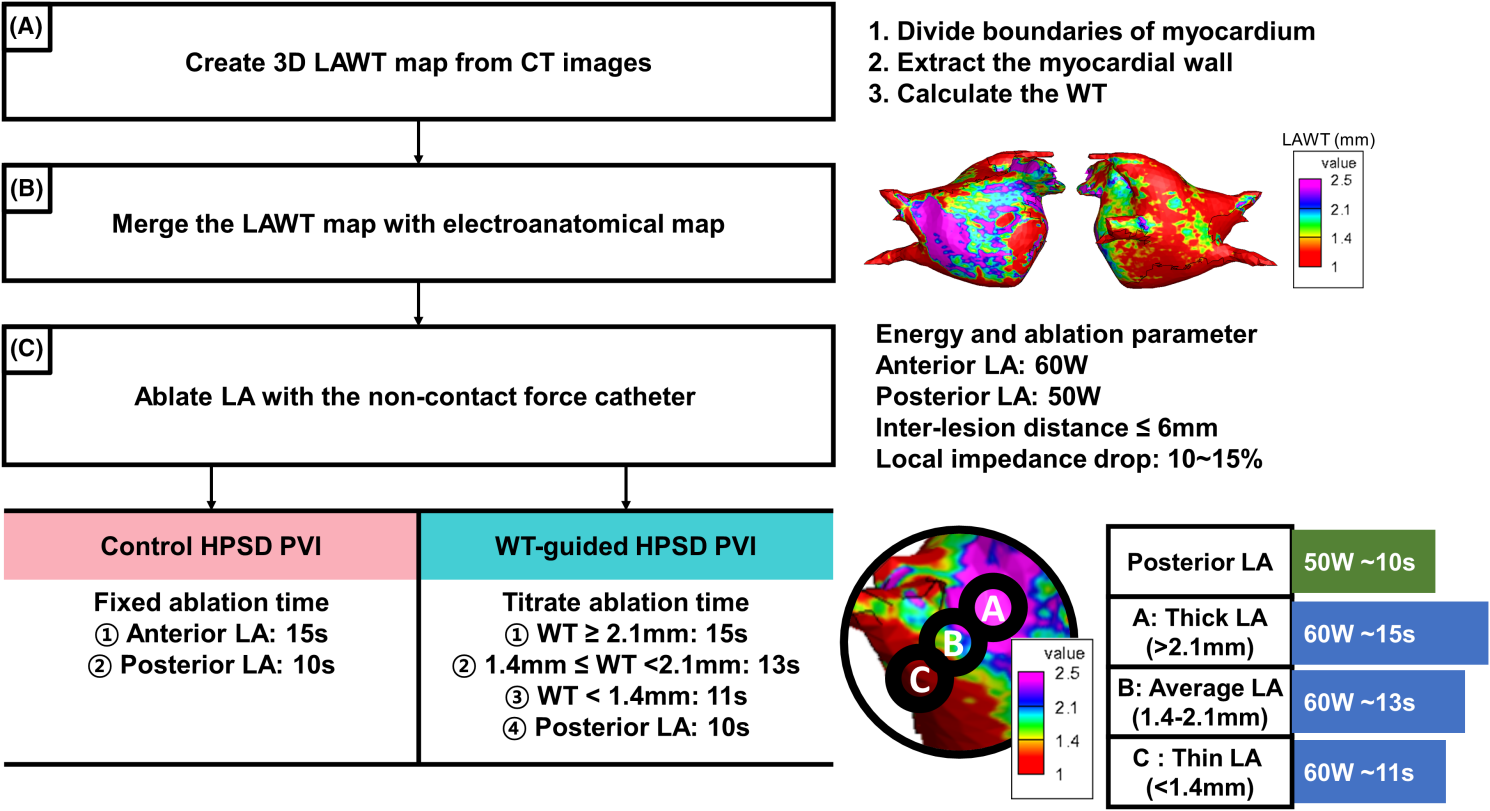
The representative images of the atrial wall thickness map and ablation protocol according to ablation strategy. (A) Using the CT images of patients, a 3D LAWT map is created in three steps. (B) The generated 3D LAWT map is merged with the electroanatomical map obtained during the procedure and is displayed in different colors based on WT values. (C) Ablation of the left atrium is performed using a non‐flexibility catheter. During this process, the anterior left atrium is ablated uniformly at 60 W, and the left posterior left atrium at 50 W, with a target inter‐lesion distance of less than 6 mm and local impedance drop between 10% and 15%. The control group ablates the anterior left atrium for up to 15 s and terminates ablation in the posterior left atrium within 10 s. The WT group performs ablation for up to 15 s for thick LA (WT above 21 mm), up to 13 s for average LA (between 1.4 and 2.1 mm), and up to 11 s for thin LA (below 1.4 mm). CT, computed tomography; HPSD, high‐power short duration; LA, left atrium; LAWT, left atrial wall thickness; PVI, pulmonary vein isolation; WT, wall thickness.

Before the ablation procedure, patients got a three‐dimensional cardiac CT scan (Philips Brilliance 63, Amsterdam, Netherlands). The CT images had spatial resolutions between 0.3 and 0.55 mm for the *x*‐ and *y*‐axes and a 0.5 mm slice thickness for the z‐axis, without overlaps or gaps. Myocardial thickness was measured using previously developed automated customized software (AMBER, LaonMed, Korea) from cardiac CT images.^15^ Obtaining myocardial wall thickness consisted of three steps; dividing the boundaries of the myocardium, extracting the myocardial wall, and calculating the wall thickness. (Figure 2) Although the structure of atria is complex, this method is robust for measuring WT owing to considering both endo‐ and epicardial surfaces.^16^ On the day before the procedure, experienced investigators in our laboratory created a 3D LAWT map, as mentioned above.

### 2.3 Merging the LAWT map with electrophysiological mapping and AFCA

Intracardiac electrograms were recorded using the Prucka CardioLab™ Electrophysiology system (General Electric Medical Systems, Inc., Milwaukee, WI, USA). We generated 3D electroanatomical maps (NavX, Abbott, Inc., USA) using the circumferential PV‐mapping catheter (AFocus, Abbott, Inc., USA; Lasso, Biosense‐Webster Inc., Diamond Bar, USA) through a long sheath, and then merged them with 3D spiral CT images. We performed transseptal punctures and obtained multi‐view pulmonary venograms for excellent matching of the 3D map, CT, and fluoroscopy. Systemic anticoagulation was initiated immediately after the septal puncture with an intravenous bolus of heparin 200 IU/kg. Intermittent heparin boluses were injected to sustain intraprocedural activated clotting time (ACT) of 350–400 s. We monitored esophageal temperature, ensuring it remained below 38.4°C during AFCA.

We used a FlexAbility catheter (Abbott Inc., USA.) without contact force monitoring in both groups. (Figure 2) The ablation points were marked automatically with contiguous RF lesions by targeting an interlesion distance (ILD) ≤6 mm and a local impedance drop of 10%–15% from baseline. Based on our previous ablation experience, 15 s was the maximum time to achieve sufficient transmural lesion formation for 60 W RF energy delivery to the anterior LA. Therefore, in the control group, we delivered a constant 60 W (for a target temperature of 45°C) up to 15 s to the anterior part of the LA and 50 W for less than 10 s to the left posterior LA with esophageal temperature monitoring to avoid esophageal injury. However, we reduced and titrated the RF delivery time according to the LAWT in the WT group. We delivered 60 W RF up to 15 s at each point with a LAWT more than 2.1 mm, 13 s at points with a LAWT around 1.4–2.1 mm, and 11 s at areas with a LAWT less than 1.4 mm. For safety purposes, we ablated the left posterior part of the LA with the same catheter (FlexAbility), power (50 W), and duration (< 10 s).

Cavotricuspid isthmus (CTI) ablation and superior vena cava (SVC) isolation were performed in most patients. Finally, we conducted high‐current burst pacing and isoproterenol provocation tests to induce extra PV triggers in both groups. If AF did not recur within 10 min of the isoproterenol provocation test, the procedure was terminated. However, if sustained AF triggers were observed, we conducted 3D activation mapping using a multielectrode catheter to locate the extra‐PV triggers. After careful mapping, we ablated extra PV triggers if possible.

### 2.4 Post‐ablation management and follow‐up

After the AFCA, fluoroscopic images were acquired, and the cardiac contours were checked for complications, including pericardial tamponade. If the patient had hemodynamic instability with pericardial tamponade, bedside echocardiography and emergent pericardiocentesis were performed. Patients who underwent pericardiocentesis remained in the hospital until the complications resolved. Patients had clinic visits at 1, 3, and 6 months, and then every 6 months. During each visit, they underwent electrocardiograms (ECGs), and 24‐h Holter recordings were obtained at the 3 months after AFCA. When the patient showed any symptom suggestive of arrhythmia recurrence within the clinical study period, the patient visited the outpatient clinic, and we took 24‐h Holter or event monitor recordings.

### 2.5 Study endpoints

We defined recurrence as any episode of AF or AT lasting at least 30 s observed on a 12‐lead ECG or Holter ECG. We classified the recurrence within 3 months of the procedure as early recurrence and the recurrence after 3 months as clinical recurrence. The primary study endpoints were the absence of clinical recurrence and the periprocedural complication rate. Secondary endpoints were procedure time, response to AADs, and electrical cardioversion rates after post‐procedural recurrences.

### 2.6 Data analysis

The sample size was estimated based on the recurrence rate derived from our previous AF ablation data.^13^ For a difference in favor of a WT‐guided RF energy titration strategy of 20%, an overall sample size of more than 162 was required to be 80% sure that the upper limit of a two‐sided 95% CI would exclude a difference in both groups. Continuous variables are presented as means ± standard deviations and assessed using the Student's *t*‐test. Categorical variables are reported as a percentage of the total group and compared using the appropriate chi‐square test or Fisher's exact test. Kaplan–Meier analysis with a log‐rank test was employed to calculate AF recurrence‐free survival and compare recurrence rates across the groups. To identify the predictors of clinical recurrence, we performed a multivariable Cox regression analysis. All variables with *p*‐values below .10 in the univariate analysis and gender were encompassed in the multivariable Cox regression analysis. We defined statistical significance as a two‐sided *p*‐value of less than .05. All statistical analyses were performed using R version 3.6.0 (R Foundation for Statistical Computing, Boston, Massachusetts, USA).

## 3 RESULTS

### 3.1 Patient characteristics

Table 1 summarizes the baseline clinical characteristics of the 212 patients enrolled in the WT (*n* = 108) and control (*n* = 104) groups, showing that both groups were well‐balanced. The mean age of the patients was 59.5 ± 11.0 years, and 145 (68.4%) were men. The mean CHA_2_DS_2_‐VASc score was 1.5 ± 1.3. Of the 212 participants, 14.2%^30^ had a history of heart failure, 29.7% (63) had hypertension, and 14.6%^31^ had diabetes mellitus. No significant differences in comorbidities were found between the two groups. The mean LA dimension was 38.1 ± 5.4 mm, and the mean left atrial volume index was 34.3 ± 9.3 mL/m^2^. We found no significant differences in the echocardiographic parameters between the two groups. In addition, there was no significant difference in regional mean WT between the WT and control groups.

**TABLE 1:** Baseline clinical characteristics.

	Overall	WT	Control	*p*‐value
	(*n* = 212)	(*n* = 108)	(*n* = 104)	
Age, (years)	59.5 ± 11.0	60.0 ± 10.2	59.0 ± 11.7	0.512
Male, *n* (%)	145 (68.4)	72 (66.7)	73 (70.2)	0.686
AF duration, (months)	31.1 ± 33.4	34.8 ± 36.7	27.1 ± 29.3	0.093
BMI (kg/m^2^)	24.2 ± 2.6	24.3 ± 2.5	24.1 ± 2.6	0.554
Comorbidities, *n* (%)				
Heart failure	30 (14.2)	16 (14.8)	14 (13.5)	0.932
Hypertension	63 (29.7)	33 (30.6)	30 (28.8)	0.903
Diabetes mellitus	31 (14.6)	18 (16.7)	13 (12.5)	0.507
Stroke	15 (7.1)	7 (6.5)	8 (7.7)	0.940
Vascular disease	10 (4.7)	6 (5.6)	4 (3.8)	0.793
CHA_2_DS_2_‐VASc score	1.5 ± 1.3	1.5 ± 1.3	1.5 ± 1.3	0.915
H2FPEF score (*n* = 190)	4.9 ± 1.3	5.0 ± 1.3	4.8 ± 1.4	0.294
Echocardiography				
LA dimension, mm	38.1 ± 5.4	38.2 ± 5.1	38.0 ± 5.8	0.804
LA volume index, mL/m^2^	34.3 ± 9.3	34.6 ± 9.8	34.0 ± 8.8	0.624
LV ejection fraction, %	65.2 ± 7.3	65.7 ± 7.6	64.7 ± 7.0	0.357
E/Em (*n* = 205)	10.0 ± 4.1	10.3 ± 4.7	9.6 ± 3.4	0.293
LV mass index, g/m^2^ (*n* = 210)	92.5 ± 19.9	95.0 ± 19.3	89.9 ± 20.3	0.064
Mean LA wall thickness, mm (*n* = 199)	1.7 ± 0.3	1.7 ± 0.3	1.7 ± 0.3	0.817
Anterior RSPV, mm	1.81 ± 0.60	1.80 ± 0.59	1.83 ± 0.61	0.720
Posterior RSPV, mm	1.33 ± 0.34	1.34 ± 0.33	1.32 ± 0.34	0.694
Anterior RIPV, mm	1.79 ± 0.60	1.78 ± 0.61	1.80 ± 0.60	0.869
Posterior RIPV, mm	1.29 ± 0.31	1.31 ± 0.31	1.27 ± 0.31	0.337
Anterior LSPV, mm	2.67 ± 0.90	2.74 ± 1.00	2.58 ± 0.76	0.217
Posterior LSPV, mm	1.30 ± 0.33	1.29 ± 0.32	1.32 ± 0.33	0.418
Anterior LIPV, mm	2.33 ± 0.69	2.31 ± 0.73	2.36 ± 0.65	0.597
Posterior LIPV, mm	1.48 ± 0.36	1.48 ± 0.36	1.47 ± 0.36	0.779
Atrial EAT volume, mL (*n* = 192)	47.3 ± 17.6	47.2 ± 16.6	47.5 ± 18.8	0.910
Mean LA voltage, mV (*n* = 198)	1.9 ± 0.8	1.9 ± 0.8	1.9 ± 0.7	0.995
LA pressure, mmHg				
Peak pressure, AF (*n* = 73)	25.0 ± 7.3	25.0 ± 7.2	24.9 ± 7.6	0.979
Mean pressure, AF (*n* = 73)	13.2 ± 5.3	13.4 ± 5.6	12.9 ± 5.0	0.703
Peak pressure, SR (*n* = 209)	21.4 ± 9.7	21.7 ± 6.6	21.2 ± 12.1	0.728
Mean pressure, SR (*n* = 209)	10.6 ± 4.8	11.1 ± 4.5	10.1 ± 5.0	0.122

Abbreviations: AF, atrial fibrillation; BMI, body mass index; EAT; Atrial epicardial adipose tissue; E/Em, ratio of the early diastolic mitral inflow velocity (E) to the early diastolic mitral annular velocity (Em); LA, left atrium; LV, left ventricle; LVMI, left ventricular mass index; LSPV, left superior pulmonary vein; LIPV, left inferior pulmonary vein; RSPV; right superior pulmonary vein; RIPV, right inferior pulmonary vein; SR, sinus rhythm.

### 3.2 Procedural characteristics

**FIGURE 3:**
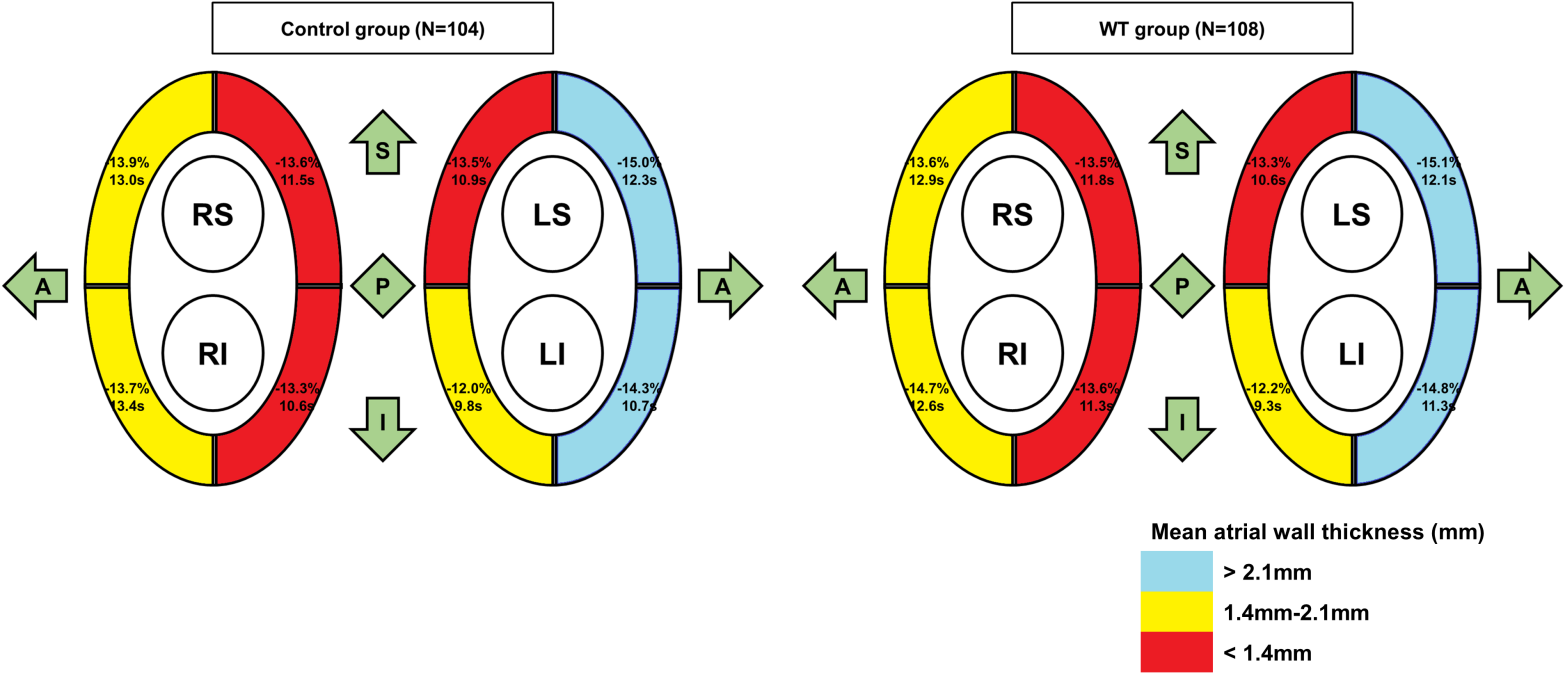
The mean wall thickness (mm), impedance drop (%), and ablation time (s) per point according to the segments between the two groups. LIPV, left inferior pulmonary vein; LSPV, left superior pulmonary vein; RIPV, right inferior pulmonary vein; RSPV, right superior pulmonary vein; WT, wall thickness.

The procedural results and clinical outcomes are summarized in Tables 2 and 3, respectively. There were no significant differences between the WT and control groups regarding the total procedure time (102.3 ± 17.3 vs. 103.4 ± 21.2 min, respectively; *p* = .657), the total ablation time (1862.5 ± 325.1 vs. 1840.3 ± 348.4 s, respectively; *p* = .632), or the first‐pass isolation (FPI) rate (50.9% vs. 55.4%, respectively; *p* = .606). Furthermore, the mean ablation time and the mean impedance drops for each point in each region between the two groups did not significantly differ in Figure 3. Post‐PVI isoproterenol provoked extra‐PV triggers in both groups (9.4% in the WT group vs. 10.8% in the control group; *p* = .926), and the locations of the extra‐PV triggers are summarized in Table 2.

**TABLE 2:** Procedural findings and characteristics.

	Overall	WT	Control	*p*‐value
	(*n* = 212)	(*n* = 108)	(*n* = 104)	
Procedure time, min	102.8 ± 19.3	102.3 ± 17.3	103.4 ± 21.2	0.657
Fluoroscopic time, min	20.4 ± 5.6	19.9 ± 5.3	21.0 ± 6.0	0.173
Ablation time, s	1851.6 ± 336.1	1862.5 ± 325.1	1840.3 ± 348.4	0.632
Mean ablation time per points, s				
Anterior RSPV	13.0 ± 3.0	12.9 ± 2.8	13.0 ± 3.2	0.731
Posterior RSPV	11.7 ± 2.7	11.8 ± 2.6	11.5 ± 2.8	0.416
Anterior RIPV	13.0 ± 3.5	12.6 ± 3.3	13.4 ± 3.6	0.101
Posterior RIPV	11.0 ± 4.1	11.3 ± 4.2	10.6 ± 4.1	0.186
Anterior LSPV	12.2 ± 2.4	12.1 ± 2.4	12.3 ± 2.4	0.502
Posterior LSPV	10.7 ± 2.5	10.6 ± 2.5	10.9 ± 2.5	0.327
Anterior LIPV	11.0 ± 2.7	11.3 ± 2.6	10.7 ± 2.8	0.159
Posterior LIPV	9.6 ± 2.4	9.3 ± 2.2	9.8 ± 2.7	0.135
Ablation lesions, *n* (%)				
CPVI	212 (100.0)	108 (100.0)	104 (100.0)	>0.999
CTI	210 (99.1)	106 (98.1)	104 (100.0)	0.494
SVC	212 (100.0)	108 (100.0)	104 (100.0)	>0.999
First pass PVI rate, *n* (%) (*n* = 209)	111 (53.1)	55 (50.9)	56 (55.4)	0.606
Extra PV triggers, *n* (%) (*n* = 208)	21 (10.1)	10 (9.4)	11 (10.8)	0.926
Coronary sinus	6 (2.9)	3 (2.8)	3 (2.9)	
Crista terminalis	1 (0.5)	0 (0.0)	1 (1.0)	
Fossa ovalis	1 (0.5)	0 (0.0)	1 (1.0)	
Septum	3 (1.4)	3 (2.8)	0 (0.0)	
LA posterior wall	1 (0.5)	1 (0.9)	0 (0.0)	
SVC	3 (1.4)	2 (1.9)	1 (1.0)	
Unmappable, multifocal	6 (2.8)	1 (0.9)	5 (4.9)	
Mean impedance drops per points (%)				
Anterior RSPV	13.8 ± 2.2	13.6 ± 2.3	13.9 ± 2.1	0.427
Posterior RSPV	13.5 ± 2.3	13.5 ± 2.2	13.6 ± 2.4	0.711
Anterior RIPV	14.2 ± 8.6	14.7 ± 11.8	13.7 ± 2.6	0.415
Posterior RIPV	13.4 ± 2.6	13.6 ± 2.9	13.3 ± 2.4	0.451
Anterior LSPV	15.0 ± 2.5	15.1 ± 2.4	15.0 ± 2.7	0.789
Posterior LSPV	13.4 ± 2.5	13.3 ± 2.6	13.5 ± 2.4	0.491
Anterior LIPV	14.5 ± 3.1	14.8 ± 2.9	14.3 ± 3.2	0.205
Posterior LIPV	12.1 ± 2.3	12.2 ± 2.4	12.0 ± 2.3	0.650

Abbreviations: CPVI, circumferential pulmonary vein isolation; CTI, cavotricuspid isthmus; LA, left atrial; LIPV, left inferior pulmonary vein; LSPV, left superior pulmonary vein; PV, pulmonary vein; PVI, pulmonary vein isolation; RA, right atrium; RIPV, right inferior pulmonary vein; RSPV; right superior pulmonary vein; SVC, superior vena cava.

**TABLE 3:** Clinical rhythm outcomes and safety.

	Overall	WT	Control	*p*‐value
	(*n* = 212)	(*n* = 108)	(*n* = 104)	
Follow‐up duration (months)	13.4 ± 7.0	13.9 ± 6.7	13.0 ± 7.4	0.368
Post‐ABL medication				
ACEi, or ARB, *n* (%)	52 (24.5)	30 (27.8)	22 (21.2)	0.337
Beta blocker, *n* (%)	75 (35.4)	36 (33.3)	39 (37.5)	0.624
Statin, *n* (%)	100 (47.2)	54 (50.0)	46 (44.2)	0.482
AAD use				
AADs at discharge, *n* (%)	38 (17.9)	20 (18.5)	18 (17.3)	0.960
AADs after 3 months, *n* (%)	44 (21.8)	28 (26.7)	16 (16.5)	0.114
AADs at final follow‐up, *n* (%)	42 (19.8)	27 (25.0)	15 (14.4)	0.079
Early recurrence, *n* (%)	35 (16.5)	23 (21.3)	12 (11.5)	0.084
Recurrence type AF, *n* (% in early recur)	26 (74.3)	17 (73.9)	9 (75.0)	>0.999
Recurrence type AT, *n* (% in early recur)	9 (25.7)	6 (26.1)	3 (25.0)	>0.999
Clinical recurrence, *n* (%)	21 (9.9)	15 (13.9)	6 (5.8)	0.080
AF Recurrence, *n* (% in recur/% overall)	18 (85.7/8.5)	12 (80.0/11.1)	6 (100.0/5.8)	0.622
AT Recurrence, *n* (% in recur/% overall)	3 (14.3/1.4)	3 (20.0/2.7)	0 (0/0)	0.622
Cardioversion, n (% in recur/% overall)	2 (9.5/0.9)	2 (13.3/1.9)	0 (0/0)	0.906
Complications				
Pericardial tamponade, *n* (%)	9 (4.2)	5 (4.6)	4 (3.8)	>0.999

Abbreviations: AAD, antiarrhythmic drug; ABL, ablation; ACEi, angiotensin‐converting enzyme inhibitor; AF, atrial fibrillation; ARB, angiotensin receptor blocker; AT, atrial tachycardia; SR, sinus rhythm.

### 3.3 Primary outcomes

**FIGURE 4:**
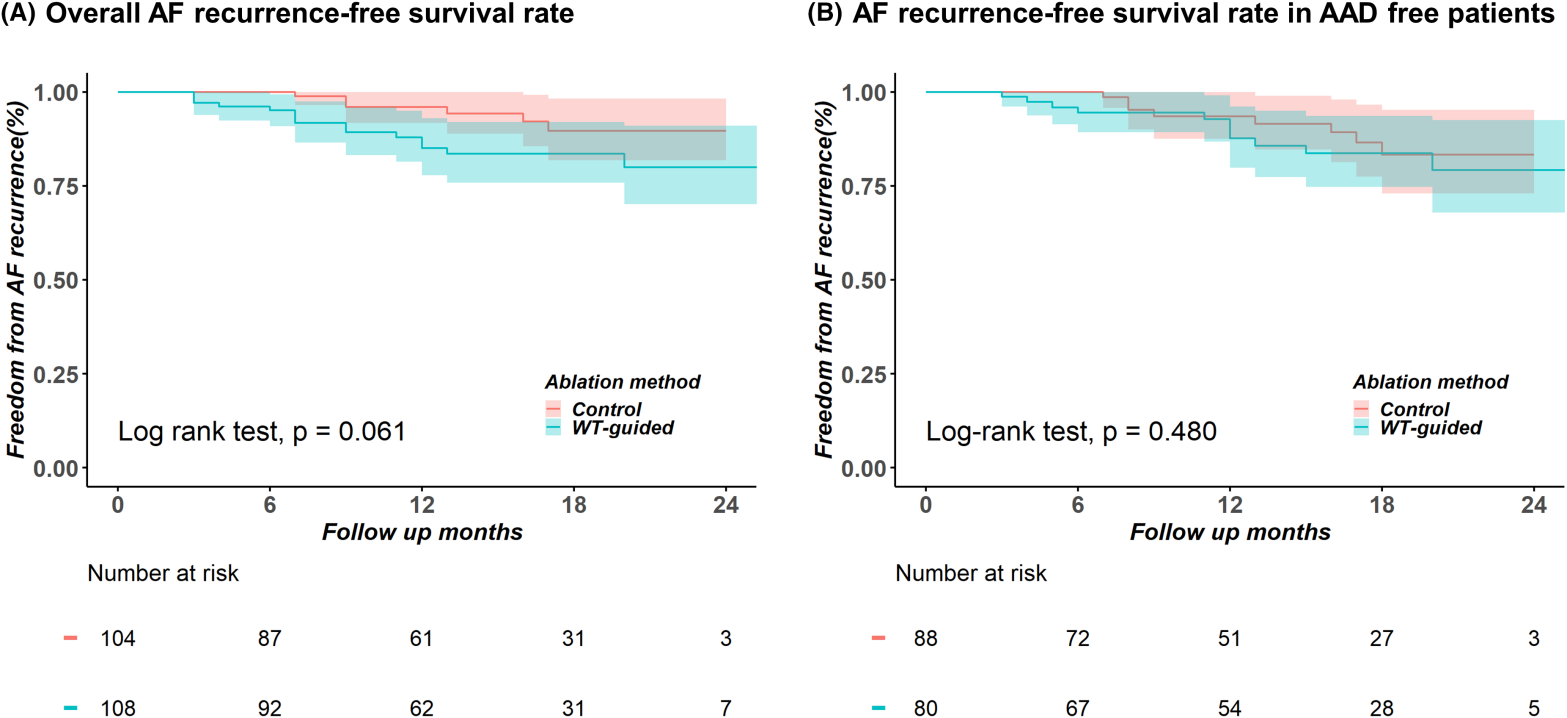
Kaplan–Meier analysis of the AF recurrence‐free survival rate (A), AF recurrence‐free survival rate in AAD‐free patients (B). AAD, anti‐arrhythmic drug; AF, atrial fibrillation.

During the mean 13.4 ± 7.0 months of follow‐up, neither the early recurrence rate (21.3% in the WT group vs. 11.5% in the control group; *p* = .084) nor the clinical recurrence rate (13.9% in the WT group vs. 5.8% in the control group; *p* = .080) significantly differed between the two groups (Table 3). The Kaplan–Meier analysis revealed no significant difference in overall AF recurrence (log‐rank: *p* = .061; Figure 4A) or AAD‐free AF recurrence (excluding patients treated with an AAD in the third month from the AFCA) (log‐rank: *p* = .480; Figure 4B) between the two groups. In the multivariable Cox regression analysis (Table 4), extra‐PV triggers (hazard ratio: 6.29 [1.77–22.38]; *p* = .005) were independently associated with the clinical recurrence of atrial arrhythmia.

**TABLE 4:** Cox regression analysis for clinical recurrence.

	Univariate		Multivariable	
	HR (95% CI)	*p*‐value	HR (95% CI)	*p*‐value
Age, years^a^	1.06 (1.01–1.11)	0.017	1.07 (1.00–1.15)	0.059
Male, *n* (%)^a^	0.75 (0.31–1.82)	0.533	1.14 (0.35–3.69)	0.833
AF duration, months	1.00 (0.99–1.01)	0.969		
Comorbidities				
Heart failure^a^	2.49 (0.97–6.43)	0.059	2.36 (0.62–6.94)	0.206
Hypertension	0.90 (0.35–2.33)	0.832		
Diabetes mellitus	0.72 (0.12–2.29)	0.397		
Stroke^a^	1.65 (0.95–2.84)	0.073	4.83 (0.96–24.17)	0.055
Vascular disease	0.38 (0.19–10.95)	0.714		
CHA_2_DS_2_‐VASc score^a^	1.36 (1.02–1.81)	0.038	0.68 (0.37–1.26)	0.218
H2FPEF score	1.25 (0.90–1.73)	0.189		
Echocardiographic parameters				
LA dimension, mm^a^	1.08 (0.99–1.18)	0.069	1.06 (0.94–1.19)	0.321
LA volume index, mL/m^2^	1.03 (0.99–1.08)	0.102		
LV ejection fraction, %	1.04 (0.97–1.11)	0.241		
E/Em	1.03 (0.95–1.11)	0.489		
LVMI, g/m^2^ ^a^	1.02 (1.00–1.04)	0.100	1.01 (0.99–1.04)	0.365
Mean LA wall thickness, mm^a^	0.32 (0.08–1.24)	0.098	0.34 (0.07–1.66)	0.181
Extra‐PV trigger, *n* (%)^a^	2.87 (0.96–8.58)	0.059	6.29 (1.77–22.38)	0.005
WT guided ablation, *n* (%)^a^	2.40 (0.93–6.19)	0.070	2.13 (0.81–5.62)	0.127

Abbreviations: AF, atrial fibrillation; CI, confidence interval; E/Em, mitral inflow velocity/mitral annulus tissue velocity; HR, hazard ratio; LA, left atrium; LV, left ventricle; LVMI, LV mass index; PV, pulmonary vein; WT, wall thickness.

^a^ Selected variables were used for multivariate cox regression analysis.

The major complication rate did not significantly differ between the two groups (4.6% in the WT group vs. 3.8% in the control group; *p* > .999). We performed pericardiocentesis right after the ablation procedure in all cases of pericardial tamponade. Eight of them were observed for an extended period of hospitalization of 1–2 days and were discharged safely. In one case of pericardial tamponade, we performed the primary repair of the right atrial free wall and discharged the patient safely after 7 days.

### 3.4 Secondary outcomes

The AAD prescription rates in the WT and control groups did not significantly differ at discharge (18.5% vs. 17.3%, respectively; *p* = .960), 3 months after the procedure (26.7% vs. 16.5%, respectively; *p* = .114), or at the final follow‐up (25.0% vs. 14.4%, respectively; *p* = .079) (Table 3). Of the 21 patients with clinical recurrence, AF recurred in 18, and AT recurred in three (Table 3). Among these patients with clinical recurrence, the proportion of AT (20.0% [3/15] in the WT group vs. 0% [0/6] in the control group; p = 0.622) and cardioversion (13.3% [2/12] in the WT group vs. 0% [0/6] in the control group; *p* = .906) did not significantly differ between the two groups.

## 4 DISCUSSION

In this prospective, single‐center, randomized clinical trial, we compared the efficacy and safety of WT‐guided RF titration by changing the energy delivery time during HPSD‐PVI. In patients with PAF, the myocardial WT‐guided PVI and conventional PVI procedures had similar efficacy during an average follow‐up of 13.4 ± 7.0 months. The clinical recurrence rate and the major complication rate did not differ significantly between the two groups. Moreover, secondary outcomes such as AT recurrence rate, cardioversion rate, and responses to AAD prescription also did not significantly differ between the two groups. However, the overall tamponade incidence rate was 4.2% and it was higher than previously reported studies.

### 4.1 Role of myocardial wall thickness in the mechanism of AF

Although several factors, such as atrial fibrosis, atrial wall stress, atrial hypertrophy, and atrial geometry, may affect AF arrhythmogenesis, we do not fully understand the effect of atrial WT on AF initiation and maintenance. Nakamura et al.^17^ reported that the LAWT was greater in patients with PAF than those with chronic atrial fibrillation or normal sinus rhythm. Takahashi et al.^18^ identified that the LA wall and PV–LA junction were significantly thicker in patients with AF than in controls. According to previous reports,^9^ an average LAWT measured by CT was a reasonable value in this study (1.7 ± 0.3 mm).

Although the causal relationship between AF and atrial WT remains unclear, there is some evidence that myocardial thickness may affect the arrhythmogenesis of AF. The theory of double layer proposes that AF remains sustained through the occurrence of endo‐epicardial breakthroughs. These breakthroughs continuously generate wavelets that emerge at the opposing layer, driven by endo‐epicardial dissociation.^19^ Transmural discordant activation plays a role in AF maintenance and re‐entrant drivers, especially pronounced in thicker parts of the atrial wall.^20^ Ravelli et al.^21^ described that the deeper penetration and slow decay of fibrosis in the subepicardial and mid‐wall layers in dilated atria and patients with atrial fibrillation may cause the formation of a 3D arrhythmogenic substrate.

### 4.2 Myocardial WT and durable PVI

Durable PVI is the cornerstone of ablation procedures to prevent atrial arrhythmias and AF recurrence.^22^ There are several reports that the WT of PV–LA junctions and their heterogeneity affect dormant PV conduction and AF recurrence.^18^, ^23^ Sciacca et al.^24^ reported a cardiac magnetic resonance assessment 3 months after very‐HPSD ablation (vHPSD, 90 W for 4 s). The right‐sided FPI was achieved significantly more often (*p* = .01) than left‐sided isolation, indicating that a thicker atrial wall around the left ridge may require more RF applications. Motoike et al.^25^ measured atrial WT using intracardiac echocardiography and compared the tailored ablation index and fixed force‐time integral protocol. They tailored the ablation index protocol and improved the FPI rate and 1‐year rhythm outcomes. Teres et al.^10^ demonstrated that modifying the ablation index for LAWT led to a decrease in RF delivery time, fluoroscopy usage, and procedure duration, all while maintaining comparable efficacy. Lee et al.^26^ showed the efficacy of the tailored ablation index based on the LAWT to reduce acute PV reconnection after PVI.

Our study is the first to evaluate the efficacy and safety of WT‐guided PVI using 60 W HPSD ablation. However, the WT‐guided energy titration did not affect the clinical outcomes of HPSD‐PVI. The main factor was that despite implementing a different ablation strategy, the reduction in ablation time (2 or 4 s) did not significantly impact the segment‐specific average ablation time and impedance drop. This aspect likely contributed to the difficulty in observing differences in the primary outcomes.

### 4.3 Risk factors for pericardial tamponade during HPSD

The overall cardiac tamponade rate in this study was 4.2%, and it was higher than 0.6%–1.5% in previous studies.^13^, ^27^, ^28^ Some studies proposed that utilizing a contact force catheter may decrease the risk of cardiac perforation^29^ and major complications.^30^, ^31^ Conversely, other studies indicated a potential rise in tamponade risk.^32^ Additional research is crucial for a comprehensive understanding. In a retrospective study conducted at our center, we previously reported^11^ a higher incidence of pericardial tamponade in female and elderly patients with thin LAWT. In this current study, when comparing the nine cases of pericardial tamponade with the 203 cases without such complications, those who experienced tamponade were older (68.7 ± 6.4 vs. 59.1 ± 10.9, *p* = .010). However, the procedural characteristics, including the mean ablation time or impedance drops for each segment between the patients with or without tamponade, did not significantly differ (Figure S1). In the multivariable regression analysis regarding the occurrence of pericardial tamponade, factors such as sex, BMI, AF duration, CHA2DS2‐VASc score, and dimension of the left atrium did not show significant differences. (Table S2) However, older age (OR 1.17, 95% CI [1.04–1.36], *p* = .022) was identified as a significant contributor. Furthermore, higher body mass index and lower epicardial adipose tissue were associated with pericardial tamponade in specific regression models. It implied that even with the same thickness of the left atrial wall, the likelihood of tamponade increased in older patients with lower epicardial adipose tissue. Zipse et al.^33^ reported that epicardial adipose tissue attenuated RF delivery and reduced the lesion depths and volumes. Nakagawa et al.^34^ reported that high tissue temperature was maintained for 12–15 s after stopping HPSD RF delivery, which may be a possible reason for frequent complications. Thus, there is a possibility that the HPSD 15 s ablation energy was excessive for PV–LA transmural lesion formation, especially in older patients with lower epicardial adipose tissue. Therefore, determining an optimal RF titer for durable PVI that satisfies both safety and effectiveness requirements is essential.

### 4.4 Limitations

This study has several limitations. First, although we assumed the superior efficacy of the WT‐guided strategy for power calculation, our tailored methods were performed with a downward energy delivery time on the thinner atrium in the WT group owing to safety concerns. Based on our experience conducting 50–60 W ablations, we observed an increased risk of complications, such as steam pops, when delivering energy for over 15 s. Consequently, we decided to set the maximum ablation time to 15 s and explored alternative approaches to modify the ablation method. Calculating the sample size for a non‐inferiority trial would be more suitable for the current research purpose. Second, other lesion quality indicators, including contact force and ablation index, were not used in our study because of the uniformity of the method. Third, because we only divided the atrial wall thickness by 1.4 mm and 2.1 mm, insufficient transmural lesion formation may have occurred at a significantly thicker (>4 mm) LA. The narrow classification criteria may have led to a similar efficacy between the control and WT groups. Fourth, our patients had higher complication rates than those reported in the previous studies. It may be attributed to the non‐use of a non‐contact force catheter. Finally, ECG‐ and Holter‐based follow‐ups may miss asymptomatic arrhythmias and affect clinical outcomes.

## 5 CONCLUSIONS

Among patients with PAF, the tailored LAWT‐guided energy titration strategy did not result in improved procedural safety and efficacy compared to the conventional 50–60 W‐HPSD CPVI. Consideration is crucial for heightened cardiac tamponade risk during 50–60 W HPSD ablation. In thickness‐guided ablation, a focused strategy and the use of pertinent ablation indices are essential for optimal outcomes.

## FUNDING INFORMATION

This work was supported by the Korea Medical Device Development Fund grant [Project number 1711174471; RS‐2022‐00141473] funded by the Korea government (the Ministry of Science and ICT, the Ministry of Trade, Industry, and Energy, the Ministry of Health & Welfare, the Ministry of Food and Drug Safety). This work was also supported by the National Research Foundation of Korea (NRF) grant funded by the Korea government Ministry of Science and ICT (MSIT) (2022R1C1C1008777).

## CONFLICT OF INTEREST STATEMENT

We previously developed automated customized software (AMBER, LaonMed, Korea) to measure myocardial thickness from cardiac CT. We registered the patent for the above technology on June 2, 2022, and had the patent number (10‐2022‐0067621). The authors declare no conflict of interest, except for the patent related to wall thickness measurement software.

## ETHICS STATEMENT

The study protocol was approved by the Institutional Review Board of Yonsei University (4‐2019‐0109).

## PATIENT CONSENT STATEMENT

Written informed consent was obtained from all participants before the start of the study.

## CLINICAL TRIAL REGISTRATION

CRIS number (KCT0003747).

## Supporting information


**Data S1:** Supporting Information.

## Data Availability

The deidentified participant data will be shared on a request basis. Please directly contact the corresponding author to request data sharing.
