# Supplementary material for: Using computed tomogram atrial myocardial thickness maps in high‐power short‐duration radiofrequency pulmonary vein isolation: UTMOST AF
Source: J Arrhythm. 2024 Mar 1;40(2):267–77. doi: 10.1002/joa3.13008 (PMC10995596; doi:10.1002/joa3.13008)
Supplement: Supplementary file 1 — Data S1: Supporting Information. [file JOA3-40-267-s001.docx]

**Supplementary material**

**1. Supplementary Table S1. Inclusion and exclusion criteria**

| **Inclusion criteria** | **Exclusion Criteria** |
| --- | --- |
| 1> Patient with paroxysmal atrial fibrillation who is scheduled for ablation procedure and ≥20 and ≤80 years of age.  2> Left atrium size < 50mm.  3> Paroxysmal atrial fibrillation that is recurrence during antiarrhythmic drug treatment or is not able to use an antiarrhythmic drug.  4> Patient who is indicated for anticoagulation therapy (for prevention of cerebral infarction). | 1> Patients with persistent or permanent atrial fibrillation.  2> Atrial fibrillation associated with severe cardiac malformation or a structural heart disease that is hemodynamically affected.  3> Patients with severe renal impairment or CT imaging difficulty using contrast media  4> Patients with a past history of radiofrequency ablation for atrial fibrillation or other cardiac surgery  5> Patients with active internal bleeding  6> Patients with contraindications for anticoagulation therapy(for prevention of cerebral infarction) and antiarrhythmic drugs  7> Patients with valvular atrial fibrillation (mitral stenosis >grade 2, mechanical valve, mitral valvuloplasty)  8> Patients with a severe comorbid disease  9> Expected survival < 1 year  10> Drug addicts or alcoholics  11> Patients who cannot read the consent form (illiterates, foreigners, etc.)  12> Other patients who are judged by the principal or sub-investigator to be ineligible for participation in this clinical study |

**2. Supplementary Table S2.** Univariate and multivariable regression analysis on the occurrence of cardiac tamponade

|  | **Univariate** | | **Model 1 (N=212)** | | **Model 2 (N=184)** | | **Model 3 (N=184)** | | |
| --- | --- | --- | --- | --- | --- | --- | --- | --- | --- |
|  | **OR (95% CI)** | **P-value** | **OR (95% CI)** | **P-value** | **OR (95% CI)** | **P-value** | **OR (95% CI)** | **P-value** | |
| Age, years | 1.13 (1.03-1.25) | 0.013 | 1.17 (1.04-1.36) | 0.022 | 1.34 (1.13-1.73) | 0.006 | 1.32 (1.11-1.73) | 0.013 | |
| Male, n (%) | 0.56 (0.14-2.34) | 0.403 | 0.66 (0.12-3.35) | 0.609 | 7.16 (0.50-157.52) | 0.166 | 4.90 (0.43-74.18) | 0.212 | |
| Body mass index, kg/m^2^ | 1.05 (0.81-1.36) | 0.721 | 1.14 (0.85-1.55) | 0.384 | 1.54 (1.04-2.44) | 0.040 | 1.56 (1.06-2.44) | 0.030 | |
| AF duration, months | 1.01 (0.99, 1.02) | 0.372 | 1.01 (0.98-1.02) | 0.578 | 1.01 (0.98-1.03) | 0.573 | 1.01 (0.98-1.03) | 0.465 | |
| CHA_2_DS_2_-VASc score | 1.39 (0.86, 2.19) | 0.153 | 0.75 (0.32-1.51) | 0.463 | 0.98 (0.25-2.68) | 0.979 | 0.93 (0.24-2.61) | 0.898 | |
| Echocardiographic parameters |  |  |  |  |  |  |  |  | |
| LA dimension, mm | 1.00 (0.89-1.14) | 0.938 | 0.97 (0.85-1.11) | 0.627 | 0.91 (0.75-1.1) | 0.339 | 0.9 (0.75-1.08) | 0.277 | |
| LA volume index, ml/m^2^ | 1.00 (0.93-1.07) | 0.934 |  |  |  |  |  |  | |
| WT guided ablation, n(%) | 1.21 (0.31, 5.03) | 0.778 | 1.23 (0.29-5.5) | 0.779 | 0.47 (0.06-3.01) | 0.428 | 0.53 (0.08-3.15) | 0.485 | |
| Mean LA wall thickness, mm | 0.55 (0.06-4.69) | 0.584 |  |  | 0.12 (0.00-2.27) | 0.185 | 0.13 (0.00-2.32) | 0.188 | |
| Epicardial adipose tissue, mm | 0.97 (0.92-1.01) | 0.194 |  |  | 0.88 (0.79-0.96) | 0.013 | 0.89 (0.8-0.97) | 0.016 | |
| Average impedance drops in anterior RIPV, % | 0.73 (0.54-0.96) | 0.030 |  |  | 0.76 (0.49-1.01) | 0.196 |  |  | |
| Average impedance drops in anterior LSPV, % | 0.72 (0.52-0.96) | 0.031 |  |  |  |  | 0.87 (0.56-1.33) | 0.535 | |
| AF, atrial fibrillation; LA, left atrium; PV, pulmonary vein; WT, wall thickness; OR, odds ratio; CI, confidence interval; RIPV, right inferior pulmonary vein; LSPV, left superior pulmonary vein | | | | | | | | |  |

**3. Supplementary Figure S1.** The mean wall thickness (mm), impedance drop (%), and ablation time (second) per point according to the segments between the patients with or without tamponade


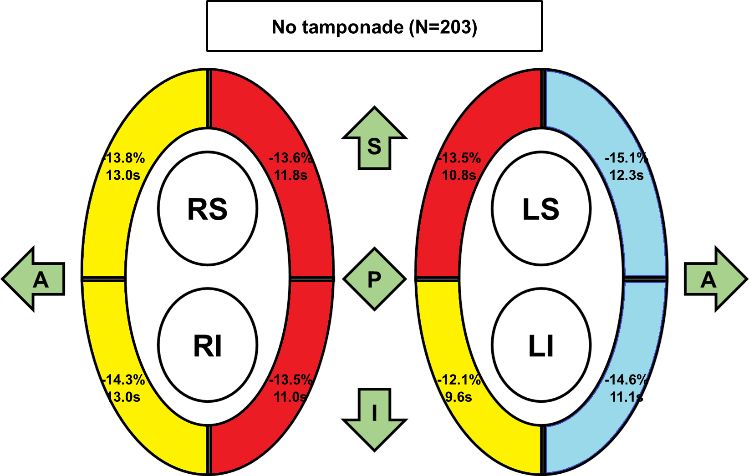

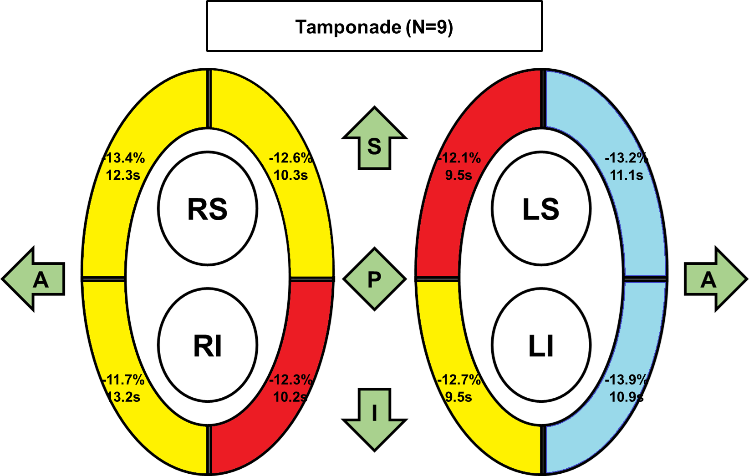
**
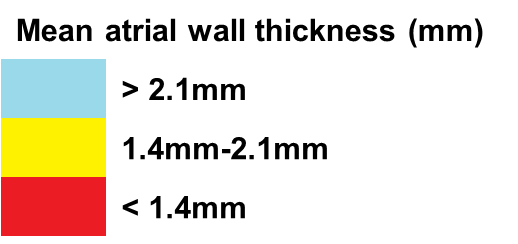
**
